# Pregnancy and parenting experiences of women with twin-to-twin transfusion syndrome: a qualitative study

**DOI:** 10.1186/s12884-021-04057-0

**Published:** 2021-09-03

**Authors:** Lijie Ren, Cancan Song, Chunling Xia, Nan Wang, Yan Yang, Shaowei Yin

**Affiliations:** 1 Gynecology and Obstetrics Department, Shengjing Hospital of China Medical University, 36 Sanhao Road, Heping District, Shenyang City, CO 110004 Liaoning Province China; 2 Nursing Department, Shengjing Hospital of China Medical University, 36 Sanhao Road, Heping District, Shenyang City, CO 110004 Liaoning Province China

**Keywords:** Twin-to-twin transfusion syndrome, Pregnancy and parenting experience, Mental state, Qualitative research

## Abstract

**Background:**

Qualitative research can reflect the actual thoughts and experience of research subjects and can be used to explore the experiences of women presenting with twin-to-twin transfusion syndrome (TTTS) to facilitate the provision of targeted psychological support.

**Methods:**

A semi-structured interview method was used to assess the pregnancy and parenting experiences of women with TTTS. Colaizzi method was used for data analysis.

**Results:**

Eighteen women participated in the study. We found that women with TTTS during pregnancy experienced persistent worry about their children’s health from the disease diagnosis to the subsequent parenting processes, even in case of minor changes in their children’s health. The lack of an efficient referral process and health information increased their uncertainty about their children’s health.

**Conclusion:**

In addition to the children’s health, other difficulties encountered during pregnancy and parenting may aggravate the pressure. Clinicians in the first-visit hospital and foetal medicine centre should improve the referral process and establish a follow-up system to provide women with health information and psychological support.

**Supplementary Information:**

The online version contains supplementary material available at 10.1186/s12884-021-04057-0.

## Background

Twin-to-twin transfusion syndrome (TTTS) is a serious complication of monochorionic diamniotic (MCDA) pregnancy. Without timely treatment, the perinatal mortality rate can reach 80–100% [1, 2]. The fetal survival rate reported after fetoscopic surgery was about 80–90% for at least one twin, and the survival rate of both foetuses was about 70%. The incidence of short-term and long-term neurological complications in surviving children was about 3–15% [3–5]. Based on the characteristics of the disease and current treatment status, women with TTTS may experience serious adverse events such as loss of the foetus, extremely premature delivery and poor foetal prognosis. Previous studies have indicated that women with TTTS may experience anxiety, depression, post-traumatic stress, and higher parenting pressure, thereby necessitating urgent clinical attention for their mental state [6–9]. However, based on current research, the lived experiences and actual thoughts during pregnancy and postpartum cannot be definitively determined, which precludes adequate provision of targeted treatments. In this regard, qualitative research is a method used to describe the life experiences of research subjects, capturing their emotions, beliefs, and behaviors. Under a framework of patient-centred treatment and care, qualitative research has received extensive attention from clinical professionals [10–12]. Otherwise, in China, although the regional fetal therapy centers have begun to establish referral networks radiated by their capabilities, it is far from forming an effective mode of rapid communication and direct referral between primary hospitals and fetal therapy centers. A large number of patients are transferred to higher hospitals through ordinary referral channels by primary hospitals, but these higher hospitals are often not fetal therapy centers and cannot complete fetal therapy. In addition, some patients were only told that they needed to visit the fetal treatment center, but they were not urged to visit the fetal therapy center in time, and they did not get enough information about fetal therapy and fetal therapy center from the primary hospitals. The above problems may cause a delay in the patient’s treatment. Our study utilized a semi-structured interview method to explore the experiences of pregnancy and parenting as well as the main difficulties encountered by women with TTTS, to develop possible solutions for improving the mental state of these women in the prenatal and postpartum periods.

## Methods

### Participants, ethics, and eligibility

This study was conducted in a foetal medicine centre of China, which is a tertiary referral centre for tens of millions of people and provides a number of fetal treatment services, including fetoscopy laser therapy for TTTS. The hospital database was searched for patients diagnosed with TTTS from January 2018 to December 2019. The diagnosis of TTTS was according to the Quintero diagnostic criteria [13]: the maximum pool depth of amniotic fluid of the recipient foetus was more than 8 cm (≥ 10 cm after 20 weeks), and the maximum pool depth of amniotic fluid of the donor foetus was less than 2 cm. All cases were in their second trimester at the time of diagnosis of TTTS. Ethical approval was obtained from the ethics committee of Shengjing Hospital of China Medical University on 18 January 2018. The study was conducted according to the principles of the Declaration of Helsinki. We contacted patients by telephone, explained the purpose of the study in detail, and invited them to participate in the study. In order to show respect and care to the participant, the interview time is determined by the participant. All the women participating in this research received an informed consent form in electronic version, we started the interview after ensuring that they understood the content of the informed consent form and obtained their electronic signature. Data collection and analysis were carried out simultaneously. When the data reached saturation, we stopped contacting eligible research subjects. And we informed them that we might quote our conversations in research results, but we concealed the information that could identify them. In research results, all quotations we used obtained participants’ agreement. Heterogeneity was noted among participants, including difference in treatment decisions and outcomes, methods of conception, and pregnancy history [14]. The exclusion criteria of this study included: one twin with malformation; Monochorionic monoamniotic twins; twin anemia-polycythemia sequence (TAPS); Selective fetal growth restriction (sFGR); patients with severe pregnancy complications; acute intrapartum TTTS; the patients not suitable for fetal therapy.

### Data collection

A semi-structured interview method was used for data collection. Participants close to the foetal medicine centre attended face-to-face interviews. Participants from other cities were interviewed by telephone. All interviews were recorded with the consent of the participants. To ensure the objectivity and truthfulness of the data, interviews were conducted by trained researchers [15, 16]. The interviews were conducted according to the following three themes: their experience following a diagnosis of TTTS, parenting experience after childbirth, and main problems encountered and help required from the time of diagnosis to the parenting period. The specific questions are presented in Supplementary file 1(English language version and the Chinese language version). And we stopped contacting qualified groups when no new themes appear during data analysis.

### Data analysis

We transcribed the recorded data within 24 h and noted the emotional state of the interviewee, including laughing, crying, pauses, and silence. After the transcription, another researcher assessed the recordings and text. Colaizzi method was used for data analysis [11]: two researchers read the text data repeatedly, extracted and coded statements related to the research purpose, and handled the different codes. Then the meaning of coding was summarized. The theme was refined, and the formation process of the theme was described in detail for the third researcher to verify. The results were then returned to the interviewee to verify the authenticity of the content [16].

## Results

### Demographics

During the study period, a total of 62 women were diagnosed with TTTS in our hospital, and 18 of them participated in the study with the age range from 24 to 39. Of the 18 participants, six attended face-to-face interviews, and 12 underwent telephone interviews. Fifteen received fetoscopy treatment, and three declined treatments. The average age of the interviewee’s newborn was 14 months. Interviewees’ pregnancy history and pregnancy outcomes are shown in Table 1.

**Table 1:** Pregnancy history and basic information of interviewees

Interviewees	Education	Marital status	Conception method	Quintero	Diagnosis of gestational weeks	Gestational weeks of delivery	Number of Surviving children	Number of adverse previous pregnancy outcome ^a^	Whether referral^b^	Interview time to delivery or miscarriage
A1	Undergraduate	married	Assisted reproduction	III	19 + 4	32	2	1	No	11 months
A2	Undergraduate	married	Natural conception	III	24	31	2	0	No	12 months
A3	Diploma	married	Natural conception	III	21 + 3	33	2	2	Yes	8 months
A4	Undergraduate	married	Natural conception	II	17 + 3	36	2	0	No	16 months
A5	Undergraduate	married	Natural conception	III	20 + 4	36	1	0	No	18 months
A6	Undergraduate	married	Natural conception	III	21 + 6	31	2	0	Yes	12 months
A7	GCSE^c^	married	Natural conception	II	22 + 1	36	1	1	Yes	13 months
A8	Diploma	married	Natural conception	III	20 + 4	34	2	1	Yes	12 months
A9	Diploma	married	Assisted reproduction	III	22 + 4	33	1	2	Yes	8 months
A10	Diploma	married	Assisted reproduction	II	23 + 1	26	0	3	Yes	8 months
A11	Undergraduate	married	Natural conception	III	24	36	2	0	No	9 months
A12	Undergraduate	married	Natural conception	III	23 + 1	27	0	1	Yes	10 months
A13	Diploma	married	Natural conception	III	21 + 3	37	1	0	No	19 months
A14	Undergraduate	married	Natural conception	III	18 + 2	34	1	1	No	20 months
A15	Diploma	married	Assisted reproduction	IV	24 + 4	33	2	2	Yes	23 months
A16	Undergraduate	married	Natural conception	III	20 + 2		0(Refuse surgery)	0	Yes	20 months
A17	Undergraduate	married	Natural conception	III	21 + 6		0(Refuse surgery)	0	Yes	19 months
A18	GCSE^c^	married	Natural conception	II	20 + 4		0(Refuse surgery)	1	No	12 months

^**a**^ number of adverse previous pregnancy outcome refers to the number of miscarriages, abortion, fetal or child deaths of the patient

^b^ Referral depends on whether the disease requires fetal therapy and whether the first hospital can provide fetal therapy

^c^*GCSE* General Certificate of Secondary Education

### Themes

After coding and analyzing the text data, three themes emerged: (1) the experiences and emotions in different periods, (2) the main barriers encountered from diagnosis to parenting, (3) required support.

### The experiences and emotion in different periods

#### Diagnosis experience

MCDA pregnancy is a high-risk pregnancy that receives more attention from clinicians than a normal single pregnancy. Although patients might be informed of potential risks of twin pregnancies, the joy of having twins was the primary mood of early pregnancy. The shock, sadness, and extreme worry about foetal health experienced by patients at the time of TTTS diagnosis showed a considerable contrast with the joy experienced in the previous period. “*The early measurements of foetuses were very normal. When the doctor told me about TTTS, I was shocked and wondered how could this happen to me? It was the first time I heard about this disease, and I thought it was very serious, I was very scared and worried tha*t both foetuses would dead”.

#### Treatment decision experience

As fetoscopy is currently the preferred treatment method for TTTS, the choice of treatment method did not bother the patients. The main issue they faced was whether to accept surgery, while family support was a critical factor in their decision. For women who opted for surgery, their primary concern was to save the foetuses. These patients believed that it was fortunate for them to have the opportunity for treatment. “*We knew the risks and prognosis of the operation, but no matter what the outcome was, we must have the operation. As long as there was a glimmer of hope, we must give the child a chance; then, we would have no regrets*”. For women who declined treatment, the decision-making process was extremely painful. After considering the potential risks, they declined surgery. Such patients typically had good fertility or already had offspring, and patients’ family members disagreed with their decision to have surgery, especially their husbands. “*During pregnancy, I accompanied fetal growth and I was very reluctant to give up my children. But I had no other choice and I could not decide on this matter alone. My family did not support me for treatment, they said that I must be responsible for my other children, and I could get pregnant again easily. We all could not accept unhealthy children*”.

#### Postoperative pregnancy experience

Monitoring was required after treatment frequently, at the same time, the patients had to bear the pressure of potential risks such as premature delivery, intrauterine death, and foetal short-term and long-term complications. Further, they faced the sudden changes in foetal health. Fourteen women out of 18 stated that their uncertainty and worry about foetal health lasted from diagnosis to delivery. Before the foetuses were born, the pressure induced by foetal health had to be borne almost entirely by themselves.

“*Surgery did not relax me too much. I was afraid that the foetuses were born too early and had sequelae. I always felt uneasy at home, because I could not see or touch them, and I did not know their state. I did not dare to move around after returning home*”. “*My family always comforted me, but I felt it did not work, I had to endure the pressure by myself*”.

The survival rate of both twins after fetoscopy is about 60%, so some women may face the risk of losing one foetus or both foetuses. The successful experience of previous fetoscopy surgery gave patients hope to give birth to two healthy children, while the sudden loss of a fetus caused a huge psychological gap for patients, leading women to worry more about the surviving fetus. For women who eventually lost two fetuses, the heavy blow meant that their previous efforts were futile. “*I was very happy that the two foetal conditions had improved at the time of the first postoperative ultrasound monitoring, but in the next monitoring, the doctor told me that the small foetus had died. I really couldn’t accept it at that time, and my mood suddenly went from heaven to hell. Although I had always known that the small foetus health was not good, I still did not want to lose it, and since then I had been worried about whether the surviving fetus would have problems*”.

“*I was so proud of this gestation. But it was really a pity that the two twins all died. All my effort for the foetuses was in vain. I did not know how to face it. I didn’t want to do and think anything at that time*”.

#### Mother-infant separation experience

Psychological stress during pregnancy ended with the successful birth of the foetuses. However, the hospitalization of the newborn led to another type of pressure on the women. During this period, they were worried about their children’s health and experienced self-blame for not taking care of their children themselves. “*It was all my fault. The children had suffered so much since birth, I didn’t fulfil my responsibility as a mother*”. *“I nearly went crazy , I could not sleep, I could not do any other things, and my heart was fully occupied by my children. I wanted to know everything about my children. I had been holing the phone and waiting for the hospital to contact me, but I was worried about receiving bad news about my children. If the children had some complications, I might not survive.* “.

#### Heavy parenting pressure

Accompanying the children throughout their growth was a positive experience overall, but women experiencing TTTS also faced greater parenting pressure. The pressure mainly comes from several aspects, including the costs of fetal therapy for TTTS, which are at patient’s expense in most parts of China; The costs other than reimbursement of neonatal medical insurance; the worries for possible neonatal hospitalization and the more serious concerns children’s health problems in the parenting process. Due to the occurrence of TTTS in the gestation, regardless of whether the children had been hospitalized after birth, these mothers said that they could not take care of them like healthy children. Because they were highly vigilant about their children’s health and were sensitized to any small abnormalities. They would pay special attention to the children’s development at each time point. If the children did not reach growth standard at a specific time, they would become anxious and look for ways to promote the child’s growth. “*When the children were just discharged, I was worried that they would suddenly stop breathing. Looking after them was like a job. I had to make sure that they had been taken care all the time, especially at night. I did not dare to sleep*”. “*Almost every month there were somethings that worried me, such as when they should look up, sit up, and stand up. If they developed later than children of the same age, I would be anxious. I would take them to the hospital and give them various dietary supplements. I would try anything to promote their growth*”.

Raising children who were diagnosed with a developmental disorder was even more troubling. The women blamed themselves for bringing the children into this world and making them suffer. Daily rehabilitation therapies slowly wore away their patience, and the uncertainty about the outcome of their children’s rehabilitation made them feel hopeless, but the love for children made them unable to give up easily. “*It was all my fault, and I became more and more suspicious of the original decision. If I had given up treatment at the beginning, the children would not suffer so much pain. Sometimes it was very annoying, why the outcome was bad after I put in so much effort. But after all, they are my own children, and I could not give up*”.

#### Long-term grief

Losing a foetus was excruciatingly painful for women experiencing TTTS. Whether they lost two foetuses after the operation or declined intervention, they would blame themselves for foetal death, and self-blame and guilt persisted for a long time after the pregnancy. “*I often had nightmares. I was sorry for my children and that I was unable to save them*”. For women who lost one foetus, they would often think of the lost foetus in the stage of raising the surviving child and loved the surviving child more passionately. “*When I see the surviving child, I often think that I once had twins, but only one survived. I felt regretful every time I thought about it. Now, I can only give more love to the living child*”.

### Main barriers

#### Lack of information at the foetal medicine centre

The treatment of TTTS was centred in the foetal medicine centre, and most patients required a referral. However, there is currently no referral process specifically for women with TTTS. Most first-visit doctors only informed them of the name of the foetal medicine centre, and patients were required to search for information about the foetal medicine centre and the referral process on their own. A substantial period of time may have been wasted in this process. Most patients thought that the process from the first-visit hospital to the foetal medicine centre was the most convoluted. They worried that the foetuses would suddenly die on the way to the centre or due to a lack of timely treatment. “*After discovering the abnormality, we drove over at night. The stress was the greatest on the way, and I cried all the way. We didn’t know the situation of foetal medicine centre, we didn’t know how to find the doctor for treatment, whether the hospital would accept me, or whether there was a chance for treatment. We were afraid that the foetuses would die on the way*”.

#### Unable to distinguish authenticity of information

As the treatment for TTTS was centred in the foetal medical centre, it was difficult for women who experienced TTTS to obtain timely information and support from the treatment hospital and first-diagnostic hospitals during pregnancy and parenting. The Internet became the main method for them to obtain health information. However, information on the Internet was complicated, and it was difficult for them to determine the authenticity of the information without professional guidance. “*I always search information from the Internet, but I did not know whether I should trust the information I found. Sometimes there were two completely different advices, and I did not know which one to trust. In order to avoid sadness, I would choose to trust the positive information*”.

#### Too much worry

Women receiving treatment would pay particular attention to foetal examination indicators and their own physiological changes after foetal surgery. For example, after obtaining colour doppler ultrasound results, they would compare the blood flow and amniotic fluid values with normal value themselves. After being discharged, they would be extremely concerned about possible contractions. Any abnormality would increase their anxiety. They knew that their worry was unnecessary and affected their lives, but no one told them what they should pay attention to. “*After the operation, I was very worried about everything about the foetus. For example, I would be anxious when I had contractions, and when there were changes in foetal movements, but I felt that the doctor did not care about these like I did. Was my worry unnecessary? I had always been afraid that the foetuses would be affected*”. The situation was the same after birth. Neonatal developmental status, lack of trace elements, and changes in the number of bowel movements would all catch their attention. They were also aware of that their anxiety might have affected their children, but due to the TTTS experience, they could not help paying attention to these details. “*I knew every detail of the children’s development. At every development point I would see whether my children had reached the standard milestones. If they did not meet the standard, I would become anxious*”.

#### The help needed

During pregnancy and parenting process, the biggest stress source for women with TTTS experience was the children’s health. We cannot easily change the health status of children, but there were things that could be done more easily that were of great help to improve their mental state throughout the whole process, such as more optimized referral process, professional guidance and psychological support.

#### More optimized referral process

Due to the lack of information on the disease, during this referral process, patients were full of fear of losing the foetuses. They hoped that the first-visit doctor could explain the basic facts of the disease, treatment status, and possibility of foetal complications during the referral. Access to reliable information could reduce the women’s anxiety and prevent blind-searching for disease information. In addition, the first-visit hospital should establish a closer relationship with the foetal medicine centre and inform patients of the treatment and referral process to help them find the treating doctor in time and alleviate their worries about treatment timing and availability. “*I hoped that the doctor would not just tell me the name of the foetal medicine centre, they could tell me how the disease would develop next. Would the foetuses die on my way to the foetal medicine centre? Would the foetal condition worsen quickly? His words determined my mentality during the referral”. “I hoped the first doctor could tell me what to do at the foetal medicine centre to find the treating doctor in time, instead of searching the treatment hospital and blindly relying on myself* “.

#### Professional guidance

Patients would encounter significant confusion in the process of postoperative pregnancy and later parenting. They hoped to contact the practitioners at the foetal medicine centre to obtain professional advice when encountering problems. They said that any form of contact, such as telephone, WeChat, or an Official Account, provided psychological support for them. “*Every time I encountered a problem, I hoped that a professional could tell me what to do. If they told me not to worry, then I would really not worry about it...especially if I trusted the professionals*”.

#### Psychological support

TTTS women described that “*On the treatment of disease and neonatal feeding, there seemed be always various complications and problems need to be dealt with*”. From discovering the disease to parenting process, they had to go through many checkpoints. They said that Doctors’ careful attention during treatment and successful treatment information obtained from talking with other patients can significantly reduce their psychological pressure. “*I hoped that the doctors and nurses could pay more attention to me, be willing to discuss the children’s situation with me and answer the confusion in my heart. This would have made me feel more relieved”.* “I especially hoped that I could discuss the children’s situation with women who had TTTS experience, so that we would provide great psychological support to each other”.

## Discussion

We found that the emotional reactions during pregnancy of women experiencing TTTS varied and their uncertainty about the children’s health persisted from diagnosis to the postpartum parenting process. Due to the influence of TTTS during pregnancy, regardless of whether the children were hospitalized after birth, these women faced great parenting pressure after birth. The lack of health information aggravated their uncertainty about the children’s health, and small abnormalities would cause them to be alert and anxious. For women who lost their children, their thoughts, guilt, and sadness for the dead children persisted for a long time.

Women who miscarried due to various reasons faced negative emotions such as post-traumatic stress and sadness for 1 year or more after delivery [17]. For women experiencing preterm birth, in addition to the negative emotional reactions mentioned above, they worried about the children’s health and faced high parenting pressure [18, 19]. Based on interview data, our study could infer that woman who encountered miscarriage or preterm birth because of TTTS would also had many negative emotions. During pregnancy, in addition to surgery, they also dealt with the fluctuation in foetal health, and their mood was calm only after a successful birth. After childbirth, patients’ concerns about their children’s health in the study often come from both TTTS itself and premature birth. Considering the complicated pregnancy and parenting experiences of such women, clinicians should pay attention to their psychological problems, especially women who lost foetuses because of TTTS or had poor fertility. Consequently, good peer support and contacting more TTTS women with good pregnancy outcome maybe was a way to improve their psychological experiences.

In addition, optimizing the referral process and providing them with healthcare guidance and reliable knowledge would reduce their psychological pressure. Previous studies have shown that women who were transferred for treatment due to premature birth had uncertainty about the foetal outcome and difficulties in adapting to the new hospital environment [20]. Our study demonstrated that due to the lack of specialized referral channels, the most significant difficulty experienced by TTTS patients was that they were required to find information about the foetal medicine centre by themselves. Due to the lack of health information from the first-diagnosing hospital, they were full of fear of the disease and uncertainty about treatment opportunities during the referral process. An efficient referral process would ensure that patients received timely treatment and get relief from anxiety during the waiting period [21, 22].Therefore, regional hospitals and foetal medicine centres should establish closer relationships, establish direct medical channels to help patients who need intrauterine treatment to find a treating doctor quickly. Before the referral, the first-diagnosing doctor should inform the patients of the characteristics of TTTS and possible disease-associated changes during the referral in order to reduce their worry about foetal death. However, for women who experienced TTTS, merely providing information support when the disease was diagnosed was insufficient to meet the patients’ needs. Women had a high demand for foetal health information during pregnancy [23, 24]. For women who experience serious medical events during pregnancy, continuous disease information and psychological support should be provided to reduce the risk of subsequent mental distress [25, 26]. Our study observed that due to cross-regional treatment, women with TTTS had difficulty obtaining postpartum healthcare information from foetal medical centres, and it was difficult for local hospitals to provide precise advice. The lack of information caused them to be at a loss when they dealt with children’s health problems. In order to provide patients with more information, the foetal medicine centre should communicate relevant disease information with the first-diagnosing hospital to improve the information education of the first-diagnosing hospital. Before patients are discharged from the foetal medicine centre, the hospital should provide sufficient disease information, so that the patient will be capable of coping with disease-associated changes. In addition, the foetal medicine centre should establish a long-term follow-up system to provide a convenient way for women with TTTS experience to contact the foetal medicine centre to address their problems.

### Advantages and disadvantages

This study utilized a semi-structured interview to explore the pregnancy and parenting experience of women with TTTS. Our results could reflect their true feelings from the diagnosis of the disease to the parenting process. However, given that patients resided in other regions, most interviews were conducted by telephone, and we could not accurately identify the emotional state of the interviewees. Furthermore, our study asked participants to recall their experiences and feelings over months or years, which could lead to recall bias. In addition, due to the small number of participants experiencing miscarriage, the research results may not fully reflect the pregnancy and postpartum life experience of these patients. Finally, some women may refuse to participate in our research because they were so sad and unwilling to recall that painful experience. This may lead our research results to be insufficiently comprehensive and rich. But in order to reduce the influence of women who refuse to participate in the study on our results, we have included more different populations as possible.

## Conclusion

Women who experienced TTTS are a high-risk population for mental distress. Factors contributing to this situation include poor medical experience, unknown neonatal prognosis and the sadness for fetal loss. Improving the referral process and establishing a follow-up system to provide such women with health information maybe support them psychologically. Future studies can explore follow-up strategies for such people and intervention methods to improve their experience.

## Supplementary Information

**Additional file 1.**

## Data Availability

The datasets generated and/or analysed during the current study are not publicly available due further research remains ongoing, but are available from the corresponding author on reasonable request.

## Abbreviations

TTTS: Twin-to-twin transfusion syndrome
MCDA: Monochorionic diamniotic
