# Supplementary material for: Pregnancy and parenting experiences of women with twin-to-twin transfusion syndrome: a qualitative study
Source: BMC Pregnancy Childbirth. 2021 Sep 3;21:595. doi: 10.1186/s12884-021-04057-0 (PMC8417964; doi:10.1186/s12884-021-04057-0)
Supplement: Supplementary file 1 — Additional file 1. [file 12884_2021_4057_MOESM1_ESM.docx]

Supplementary file 1Interview questions for women undergoing TTTS

| 1. Could you talk about your experience of diagnosis? What was the process of coming to the foetal medicine centre? Was there any way to make you feel better during the whole process? |
| --- |
| 2. How did you make your treatment decision? What were the factors that motivated you to make the decision? |
| 3. Could you talk about your life experience after surgery/induction of abortion? What were the difficulties you encountered during the period? What support did you hope to get? |
| 4. Could you talk about your experience after delivery (when the children were hospitalized and discharged)? Would you pay more attention to your children’s health? What were the sources of stress in the parenting process? Did you think there is any way to reduce your stress? |
| 5. During the whole process, what ways did you use to get disease information? How did the information affect you? Did you discuss the information with doctors? Did you judge that the information you had obtained was correct? |
| 6. Do you have anything else you want to tell us about this pregnancy? |

经历双胎输血综合征女性的访谈提纲（中文）

| 1.可以谈谈你确诊双胎输血综合征的经历吗？来胎儿医学中心的过程是怎样的？在整个过程中有什么方法可以让你好受些吗？ |
| --- |
| 2.如何做出治疗决策？促使你做出该决定的因素有哪些？ |
| 3.可以谈谈你在治疗或者引产之后的生活经历吗？这期间你遇到什么困难？你需要什么样的帮助？ |
| 4.可以谈谈你分娩之后的经历吗（包括孩子住院和出院后的经历）？你会更关注孩子的健康吗？养育孩子过程中的压力源是什么？有什么方法可以减轻你的压力？ |
| 5.在整个过程中，你怎么获取疾病信息？这些信息是如何影响你的？你会和医生讨论获取的信息吗？你怎么判断获取信息的正确性？ |
| 6.关于这次妊娠，你还有其他想说的吗？ |
